# Incidence and clinical characteristic of ocular surface manifestation: an evaluation of conjunctival swab results in Corona Virus 2019 (COVID-19) patients in Jakarta, Indonesia

**DOI:** 10.1186/s12348-023-00343-4

**Published:** 2023-04-25

**Authors:** Made Susiyanti, Hisar Daniel, Diah Faridah, Dinda Arken Devona, Pradnya Pramitha, Budiman Bela, Budi Haryanto, Julie Dewi Barliana, Dian Estu, Andi Arus Victor, Nina Dwi Putri, Julius Candra, Nathania Sutandi, Rita S. Sitorus

**Affiliations:** 1 Department of Ophthalmology, Faculty of Medicine Universitas Indonesia, Cipto Mangunkusumo National Referral Hospital, Jalan Kimia No. 8-10, Jakarta, 10320 Indonesia; 2 Department of Ophthalmology, Faculty of Medicine Universitas Indonesia, Persahabatan Hospital, Jakarta, Indonesia; 3 Department of Clinical Microbiology, Faculty of Medicine Universitas Indonesia, Cipto Mangunkusumo National Referral Hospital, Jakarta, Indonesia; 4 Department of Clinical Microbiology, Faculty of Medicine Universitas Indonesia, Persahabatan Hospital, Jakarta, Indonesia; 5 Department of Child Health, Faculty of Medicine Universitas Indonesia, Cipto Mangunkusumo National Referral Hospital, Jakarta, Indonesia

**Keywords:** COVID-19, Ocular manifestation, Conjunctival swab

## Abstract

**Objectives:**

This study aimed to investigate the spectrum of ocular characteristics and viral presence in the conjunctival swab of patients with COVID-19.

**Methods:**

In this cross-sectional study, fifty-three patients were recruited from two COVID-19 referral hospitals in Jakarta (Cipto Mangunkusumo Hospital and Persahabatan Hospital) from July 2020 to March 2021. The inclusion criteria were patients who were suspected of or confirmed cases of COVID-19 with or without ocular symptoms. Demographic data, history of COVID-19 exposure, underlying medical condition, systemic symptoms, ocular symptoms, supporting laboratory results, reverse-transcriptase polymerase chain reaction (RT-PCR) of naso-oropharyngeal (NOP) swab and conjunctival swab were collected.

**Results:**

Fifty-three patients who were suspected, probable or confirmed cases of Covid-19 were included. Forty-six out of 53 patients (86.79%) tested positive for either Covid-19 antibody rapid test or naso-oropharyngeal (NOP) swab. Forty-two patients tested positive for NOP swab. Fourteen out of 42 patients (33.33%) experienced symptoms of ocular infection including red eye, epiphora, itchy eyes, and eye discharge. None of these patients were tested positive for conjunctival swab. Two out of 42 patients (4.76%), who were tested positive for conjunctival swab, did not experience any ocular symptoms.

**Conclusions:**

Establishing the relationship between Covid-19 infection, ocular symptoms, and presence of SARS-CoV-2 virus on the ocular surface proves to be challenging. In Covid-19 patients, ocular symptoms did not warrant a positive conjunctival swab result. On the contrary, a patient without ocular symptoms can also have detectable presence of SARS-CoV-2 virus on the ocular surface.

**Supplementary Information:**

The online version contains supplementary material available at 10.1186/s12348-023-00343-4.

## Introduction

Since December 2019 until now, a new disease called Coronavirus disease 2019 (COVID-19) had emerged in Wuhan, China. The disease had quickly spread across the globe and become a pandemic that pose a highly serious threat to millions of people. The etiology of the disease is severe acute respiratory syndrome novel coronavirus 2 (SARS-CoV-2), which causes infection of the respiratory tract that can lead to multiple organ infections and result in death. It has similarities in receptors and other properties with the former member of coronavirus (SARS-CoV-1) [1–4].

SARS-CoV-2 gains entry to host cells through angiotensin-converting enzyme 2 (ACE-2) receptors, which are expressed in many organs. Conjunctival and corneal cells were also shown to have ACE-2 receptors, thus providing possible route of entry [5–7]. Previous studies have found that the SARS-CoV-2 could be detected in tear and conjunctival tissues both in the presence and absence of ocular symptoms [1–4]. In addition, the rate of positivity in ocular secretion also varied between 0 to 28% across studies [8, 9].

Several studies have investigated the correlation between SARS-CoV-2 infection and its ocular manifestations, however many results remained controversial [1–3]. The ocular manifestations reported ranged from simple conjunctivitis with symptoms of red eyes, epiphora, conjunctival secretion, and chemosis to keratoconjunctivitis, posterior uveitis, retinitis, and optic neuritis, which affect visual acuity [4–6, 10]. The prevalence of conjunctivitis in studies widely varied, ranging from 2 to 31% [7, 11, 12]. Another study also reported that 11.64% of COVID-19 cases experienced forms of ocular symptoms [6, 13].

As viral transmission is possible through body fluid and mucous membranes, the role of ocular surface and conjunctival secretion as possible reservoir and route of transmission in patients infected with COVID-19 remains under discussion [1, 2, 4, 14, 15]. Therefore, our study aimed to focus on exploring the ocular manifestations in COVID-19 patients and detecting the presence of SARS-CoV-2 in conjunctival secretion through reverse transcriptase-polymerase chain reaction (RT-PCR) procedures. To the best of our knowledge, this is the first Indonesian study, which investigated the spectrum of ocular manifestations and results of conjunctival swab in COVID-19 patients in two general hospitals in Jakarta, Indonesia.

## Methods

### Definitions

A suspect case of COVID-19 was defined as patient who has either one of the criteria below: (i) patient with acute respiratory tract infection (ARTI) who has a travel history or stay in a country or region in Indonesia which reported local transmission of the disease within 14 days, (ii) patient with signs and symptoms of ARTI who has a contact with a confirmed or probable case of COVID-19 within 14 days, (iii) patient with severe ARTI/pneumonia who needs to be hospitalized and has no other probable etiologies. A probable case of COVID-19 was defined as suspect case with severe ARTI/ARDS or death with clinical symptoms of COVID-19 and has not been confirmed with RT-PCR examination. A confirmed case of COVID-19 was defined as patient with/without symptoms of COVID-19, who has been confirmed with naso-oropharyngeal (NOP) RT-PCR examination [16, 17].

### Study design

This cross-sectional study was conducted in two COVID-19 referral hospitals in Jakarta, which are Cipto Mangunkusumo and Persahabatan Hospital. The data was collected from July 2020 to March 2021. This study has been approved by the Ethics Committee in both institutions and was conducted in accordance with the Declaration of Helsinki. Informed consent was obtained from all participants. We included patients who were hospitalized in either of these hospitals. The inclusion criteria of this study were children or adult who fulfilled the indicator of suspected cases of COVID-19, patients with positive result of COVID-19 rapid test or NOP RT-PCR test, with or without ocular symptoms. We excluded patients who were critically ill to have the examination and conjunctival swab performed. Recorded audio informed consent was obtained from patients and/or legal guardians for underaged children. This study has obtained ethical clearance from the ethics committee of the Faculty of Medicine, University of Indonesia (ethical approval number: KET-467/UN2.F1/ETIK/PPM.00.02/2020).

### Sample collection

Cases were reported by one ophthalmologist from each hospital delegated for this task. Patient history was taken including the demographic information, contact history, clinical symptoms. Ocular signs and symptoms were also recorded. Conjunctival swab were done simultaneously by the ophthalmologist following appropriate infection control and prevention measures. Conjunctival swab was used to collect the conjunctival secretion and tears from the patient. A single conjunctival swab was collected from both eyes using a sterile dacron flocked swab in the lower eyelid fornix without topical anesthesia. The swab was then broken off and placed into a viral transport media inside the sampling tube and stored in a 4 degrees Celsius environment. RT-PCR test of the conjunctival swab was performed to assess the presence of SARS-CoV-2 RNA in the sample and was processed in clinical microbiology laboratory in both hospitals.

As our study began at the early phase of the pandemic, qualitative antibody testing from blood was the only rapid test available and performed with rapid test kit for Covid-19 IgM/IgG. A total of 10µL of blood collected from finger pin-prick was added to the sample pad, then two drops of sample buffer were added. After a 10-min incubation period, results were interpreted in which the presence of only the control line was negative, the presence of the control line with IgM and/or IgG line was considered positive. NOP RT-PCR swab test was also performed in all patients. The NOP RT-PCR swab were performed based on standardized guidelines for COVID-19 diagnosis by the Indonesian Health Ministry [17].

### Data collection

We collected information on patients’ demographic (age, gender), history of contact, underlying medical condition, systemic condition (COVID-19 symptoms, body temperature), ocular manifestations (ocular symptoms, diagnosis, uncorrected visual acuity), supporting examination result, RT-PCR of NOP swab, and RT-PCR of conjunctival swab through questionnaire-styled history taking, physical examination, and patients’ medical records. The questionnaire was filled in by the ophthalmologist in charge in each hospital. The detail of the questionnaire can be seen in Appendix 1. According to guidelines by the National Institute of Health (NIH), severity of systemic symptoms of our study was classified as mild, moderate, or severe. None of the patients in this study were asymptomatic or critical. Guidelines by the NIH stated that asymptomatic or pre-symptomatic patients were those who tested positive for SARS-CoV-2 but experienced no symptoms of Covid-19. Patients with mild illness showed varying symptoms and signs of Covid 19 but without dyspnea, shortness of breath or abnormal chest imaging. Patients with moderate illness showed signs of lower respiratory infection either by physical examination or imaging, with an oxygen saturation (SpO2) ≥ 94% on room air at sea level. Patients with severe illness have SpO2 < 94% on room air at sea level, a ratio of arterial partial pressure of oxygen to fraction of inspired oxygen (PaO2/FiO2) < 300 mm Hg, a respiratory rate > 30 breaths/min, or lung infiltrates > 50%. Patients who were critical included those with respiratory failure, septic shock, and/or multiple organ dysfunction[17, 18].

### Statistical analyses

All statistical analyses were performed using SPSS software version 24.0 for Mac (SPSS Inc, Chicago, IL, USA). Categorical data were reported in the form of frequency (percentage). Continuous variables were reported in the form of mean ± SD or median (range). Normality of the data was evaluated with Shapiro–Wilk test. Independent t-test was conducted to evaluate significant difference between the groups. A P-value of less than 0.05 was considered statistically significant.

## Results

A total of 53 patients who were defined as suspect, probable or confirmed cases of Covid-19 were recruited for this study. About 46 out of 53 patients (86.79%) tested positive for either Covid-19 antibody rapid test or NOP swab, while 4 out of 13 patients with positive antibody rapid test tested negative for both NOP and conjunctival swabs. Of all 42 patients tested positive for NOP swab, two out of them (4.76%) were also tested positive for conjunctival swab test. Summary of the result was listed in Table 1. The number of days between NOP and conjunctival swabs taken ranged from 0 to 7 days with median of 2 days.

**Table 1:** Summary of Covid-19 tests result

Type of Test	No. of Patients Tested Positive
Rapid Test Only	4
Rapid Test and NOP RT-PCR	9
NOP RT-PCR Only	31
NOP and Conjunctival RT-PCR Positive	2

Forty-two patients with positive NOP swab ranged from 1 to 69 years old with a mean age of 33.69 ± 22.38 years old. Twenty-six out of 42 patients (61.9%) were male and 10 patients (38.1%) were female. A total of 30 out of 42 patients (71.4%) experienced several systemic symptoms including fever, dyspnea, cough, anosmia, myalgia, and gastrointestinal symptoms, while others remain asymptomatic. Twenty-one out of 42 patients (50%) had systemic comorbidities, with diabetes, hypertension, and coronary artery disease being the most common one. Others include hyperthyroidism, chronic kidney disease, leukemia and systemic lupus erythematosus.

Laboratory results collected, which include blood count, procalcitonin, ureum, creatinine, alanine aminotransferase (ALT), aspartate aminotransferase (AST) as well as C-reactive protein were not significantly different between patients with positive conjunctival swab versus patients with negative conjunctival swab as shown in Table 2.

**Table 2:** Comparison of laboratory results between patients with positive and negative conjunctival swab results

Variable	Positive Conjunctival Swab (*n* = 2)	Negative Conjunctival Swab (*n* = 40)	*P*-value*
Hemoglobin	13.40 ± 1.27	12.14 ± 2.69	0.656
Platelet count	173.50 ± 21.92	267.10 ± 149.87	0.164
Procalcitonin	0.21 ± 0.11	3.59 ± 11.88	0.468
Urea	37.00 ± 22.63	36.60 ± 40.05	0.790
Creatinine	1.00 ± 0.28	1.39 ± 2.61	0.519
ALT	44.50 ± 30.41	60.15 ± 127.99	0.668
AST	52.00 ± 28.28	50.57 ± 91.53	0.733
CRP	127.05 ± 72.90	57.58 ± 62.43	0.985

^*^Independent t-test

Approximately 14 out of 42 patients (33.33%) had infection related ocular complaints including red eye, epiphora and eye discharge. During physical examination, some patients also presented with palpebral edema, chemosis of conjunctiva, and decreased visual acuity. Details of the clinical and ocular characteristics of patients with positive NOP swab and infection related ocular signs and symptoms were listed in Table 3. None of the patients with ocular complaints tested positive in conjunctival swab. On the contrary, none of the two patients who tested positive in conjunctival swab experienced any ocular symptoms. Clinical characteristics of the patients who tested positive for conjunctival swab were summarized in Table 4.

**Table 3:** Clinical characteristics of patients with positive NOP RT-PCR swab result experiencing infection related ocular symptoms

Patient No	Age	Sex	Underlying medical condition	Clinical symptoms	Ocular signs & symptoms	Ocular diagnosis	Bedside UCVA	Systemic severity	NOP swab	Conjunctival swab (CS)
2	31	Female	None	None	Red eye, epiphora, and lesion in palpebral edge	Herpes zoster ophthalmicus OD	> 3/60 OD, > 3/60 ODS	Mild	Positive	Negative
3	20	Female	None	None	Red eye, epiphora	Conjunctivitis OS contact lens related, Myopia ODS	> 3/60 OD, > 3/60 ODS	Mild	Positive	Negative
4	28	Male	None	Anosmia	Epiphora	Conjunctivitis OD and dry eyes ODS	> 3/60 OD, > 3/60 ODS	Mild	Positive	Negative
8	59	Male	Diabetes, Hypertension	None	Red eye and palpebral edema	Endophthalmitis OD	Light perception with projection (LPWP) OD, 6/6 OS	Mild	Positive	Negative
9	57	Male	Diabetes, Hyperthyroidism	Fever	Red eye, palpebral edema, eye discharge, chemosis of conjunctiva	Panophthalmitis OS dd/Orbital cellulitis OS, Secondary glaucoma OS, Complicated cataract OS, Nerve III IV VI OS paresis with pupillary involvement	LPWP OS, 6/6 OD	Mild	Positive	Negative
10	55	Female	None	None	Red eye, eye discharge, itchy	Conjunctivitis OS	> 3/60 OD, > 3/60 OS	Mild	Positive	Negative
11	3	Female	None	None	Red eye	Conjunctivitis ODS, Infantile cataract OD, Aphakia post cataract extraction OS	Blink reflex + OD, Blink reflex + , Light following + , Avoidance movement + OS	Mild	Positive	Negative
13	22	Male	None	None	Red eye, reduced vision	Vitreous opacity ec susp panuveitis OD	1/300 good projection OD, 6/6 OS	Mild	Positive	Negative
33	23	Female	ROSC after severe eclampsia	Dyspnea	Red eye and lagophthalmos	Exposure keratitis ODS	> 3/60 OD, > 3/60 OS	Severe	Positive	Negative
34	48	Female	Chronic kidney disease (on dialysis), Hypertension	Fever, cough	Red eye, palpebral edema, epiphora, and conjunctival chemosis	Panophthalmitis OS, history of endophthalmitis and neovascular glaucoma OS	> 3/60 OD, NLP OS	Moderate	Positive	Negative
37	39	Male	None	None	Red eye, palpebral edema, epiphora, and conjunctival chemosis	Post repair in corneoscleral rupture OS, Traumatic cataract OS	1/300 OD, 1/300 OS	Mild	Positive	Negative
39	69	Male	None	None	Red eye and epiphora	Endophthalmitis ODS	LPWP OD, LPWP OS	Mild	Positive	Negative
49	11	Male	Moderate depression, AML, febrile neutropenia	Fever, myalgia, malaise	Red eye	Viral dd bacterial conjunctivitis OS	> 3/60 OD, > 3/60 OS	Mild	Positive	Negative
53	12	Female	End stage renal disease ec nephrotic syndrome steroid resistant	Fever, runny nose	Epiphora	None	> 3/60 OD, > 3/60 OS	Mild	Positive	Negative

**Table 4:** Clinical characteristics of patients with positive conjunctival RT-PCR results and positive NOP RT-PCR results

Patient No	Age	Sex	Underlying medical condition	Clinical symptoms	Ocular history	Ocular Signs & Symptoms	Bedside UCVA	Systemic Severity	NOP Swab	Conjunctival Swab (CS)	Difference between NOP and CS (days)
26	69	M	None	Cough, sore throat	None	None	> 3/60 OD, > 3/60 OS	Mild	Positive	Positive	1
28	52	F	None	Cough, runny nose	Myopia ODS	None	> 3/60 OD, > 3/60 OS	Mild	Positive	Positive	0 (same day)

## Discussion

The mucous membrane of the upper respiratory tract is the main portal entry of SARS-CoV-2. The severe acute respiratory syndrome coronavirus 2 (SARS-CoV-2) infection is initiated after type 2 transmembrane protease (TMPRSS2) cleaves the viral spike glycoprotein, which then binds to the angiotensin-converting enzyme 2 (ACE-2) receptor. Presence of ACE-2 receptors and TMPRSS2 in the ocular surface makes it a potential port of entry for SARS-CoV-2[19–21].

We found 2 cases of patients with positive conjunctival swabs and NOP swabs. Ocular symptoms were not observed in these patients. NOP and conjunctival swabs were taken 0 and 1 day apart. The low rate of viral detection in conjunctival samples for our study (2 out of 42 patients) with positive NOP samples (4.76%) might be due to several causes. First, majority of the patients in our sample group might be exposed to the virus via the upper respiratory tract pathway instead of the ocular surface. As the virus would descend from the upper to the lower respiratory tract, viral load was therefore hardly detected in the conjunctival samples [22–24]. The second probable reason was a fast clearance rate of the virus from the ocular surface due to drainage via the nasolacrimal pathway, evaporation, and its low volume [7, 25]. The third reason was the fact that ocular secretions were known to have a strong local immune system (lactoferrin and immunoglobulins) against coronavirus[7, 24, 25]. Lastly, the low number of ACE-2 receptors to have been shown in conjunctival tissue (< 50% lower than other tissues), thus their ability to bind to the virus is very poor [25, 26]. It is also possible that the viruses appear at specific period of time in the ocular surface, thus requiring price collection time in order to gain positive results [10, 27]. Other studies have supported these findings, reporting the low rates of positive conjunctival swab results ranged from 0 to 15.6% [1, 2, 16, 20, 28, 29]. Factors including technical errors, difficulties in sampling, handling, and processing, the stage of the disease, low sensitivity of RT-PCR, and variable time sampling may also cause variations of positivity rate [30].

Fourteen out of 42 patients experienced infection related ocular complaints including red eye, epiphora and eye discharge. However, conjunctival samples were negative for SARS-CoV-2. Only one patient, who was clinically diagnosed as Herpes Zooster Ophthalmicus, was given topical antiviral (acyclovir) after the conjunctival samples were collected. Others were prescribed topical antibiotic and/or topical anti-inflammatory eyedrops based on the findings. Symptoms were relieved and the patients recovered without ocular complications. The relatively low number of ocular manifestations in COVID-19 patients were supported also by a systematic review and meta-analysis conducted by Aggarwal et al., which reported that only 8.35–11.64% of COVID-19 patients would present with at least one ocular symptom [31].

A low percentage of patients with conjunctivitis who had positive conjunctival swab results was reported [20]. A study by Ranzenigo et al. [32], Ozturk et al. [5], and Sonmez et al. [13] revealed none of the patients in the study with ocular symptoms tested positive for conjunctival swab and tear samples. This was also supported by meta-analysis done by Aggarwal et al., which reported low positivity rate of only 3.5% in patients with or without ocular symptoms [31]. These studies along with our results suggested that the occurrence of conjunctivitis is a systemic response rather than local activity of the virus on the ocular surface [5, 10, 13].

Ocular involvement in Covid-19 patients other than conjunctivitis has been described, including keratitis, uveitis, retinal pathologies, and neuro-ophthalmological involvement [25, 32, 33]. These clinical manifestations often occur in patients who required mechanical ventilation and in patients with more severe illness [20, 34, 35]. As our patients who had positive conjunctival swab results and mild symptoms of systemic Covid-19, ocular symptoms were not observed.

Laboratory results of patients with positive conjunctival swab results was not significantly different from patients with negative conjunctival swab results as shown in Table 3. A previous study by Atum et al. also found no significant difference in laboratory result characteristics between the two groups [36]. However, this might be due to the small number of patients who had positive conjunctival swab results and might not be statistically representative of the cohort.

Viral entry through the ocular route is a cause of concern as detectable viral presence in the ocular surface is not always symptomatic and eye-hand-eye as well as hand-eye transmission are possible. It might necessitate further protective measures such as the use of eye protection in conjunction with wearing masks, googles, gloves, and face shields, especially for health workers and ophthalmologist [6, 19, 21, 22]. Viral entry into the ocular surface can be caused by direct exposure to droplets, aerosol, contact with contaminated ophthalmic instruments or hand to eye contact after touching a contaminated surface [6, 19, 23, 37].

This study also had several limitations. First, the sample size was relatively small and it also came from only two medical centers. Second, the information regarding the COVID-19 vaccination history of patients is unavailable, as the data was taken in the early phase of the pandemic, in which the vaccination policy had not yet been fully implemented in Indonesia. Third, only one sample of conjunctival swab was taken from each patient. For further studies, we suggest to do the follow-up of COVID-19 patients, isolate the live virus, quantify conjunctival viral load, and take multiple conjunctival samples at variable days in order to better understand the correlation between the presence of SARS-CoV-2 virus and ocular manifestations in COVID-19 patients.

## Conclusion

The presence of ocular symptoms might not warrant a positive conjunctival swab result while the presence of SARS-CoV-2 virus on the ocular surface might not cause ocular symptoms. The positivity rate of conjunctival RT-PCR swab was 4.76% in patients with confirmed SARS-CoV-2 infection. As ocular symptoms often occur as a systemic response rather than local viral activity, conjunctival swabs were negative for our patients who experienced symptoms of conjunctivitis. More extensive studies need to be carried out to identify the role of SARS-CoV-2 infection in the ocular surface.

## Supplementary Information

**Additional file 1: Appendix1.** COVID-19 Questionnaire Form.

## Data Availability

The datasets used and/or analyzed during the current study are available from the corresponding author on reasonable request.

## Abbreviations

COVID-19: Coronavirus disease 2019
SARS-CoV-2: Severe acute respiratory syndrome novel coronavirus 2
SARS-CoV-1: Severe acute respiratory syndrome novel coronavirus 1
RT-PCR: Reverse transcriptase-polymerase chain reaction
NOP: Naso-oropharyngeal
NIH: National Institute of Health
ALT: Alanine aminotransferase
AST: Aspartate aminotransferase
TMPRSS2: Type 2 transmembrane protease
(ACE-2) receptor: Angiotensin-converting enzyme 2
CD 147: Cluster differentiation 147
CTSL: Cathepsin L
