# Supplementary material for: Incidence and clinical characteristic of ocular surface manifestation: an evaluation of conjunctival swab results in Corona Virus 2019 (COVID-19) patients in Jakarta, Indonesia
Source: J Ophthalmic Inflamm Infect. 2023 Apr 25;13:20. doi: 10.1186/s12348-023-00343-4 (PMC10127182; doi:10.1186/s12348-023-00343-4)
Supplement: Supplementary file 1 — Additional file 1: Appendix1. COVID-19 Questionnaire Form. [file 12348_2023_343_MOESM1_ESM.docx]

**Appendix 1. COVID-19 Questionnaire Form**

| **Name of hospital**  Filled in by  Date of examination | | :  :  : |
| --- | --- | --- |
| **Subject participant**  Medical record number  Full name  Gender  Date of birth (age)  Address  Phone number  Occupation  Past medical history | | : ……………………………………………..  : ……………………………………………..  : Male / Female  : ……………………………………………..  : ……………………………………………..  : ……………………………………………..  : ……………………………………………..  : ……………………………………………..  ……………………………………………… |
|  | **Questions** | **Answer** |
| 1. | History of contact with patients diagnosed with COVID-19 | Yes / No |
| 2. | Patients experienced symptoms as follows: |  |
|  | a. Fever | Yes / No |
|  | b. Cough | Yes / No |
|  | c. Runny nose | Yes / No |
|  | d. Sore throat | Yes / No |
|  | e. Difficulty in breathing | Yes / No |
|  | f. Myalgia | Yes / No |
|  | g. Gastrointestinal symptoms | Yes / No |
|  | h. Other symptoms, please mention: | _____________________ |
| 3. | Body temperature | ___________ degrees Celcius |
| 4. | Patients experienced ocular signs and symptoms as follows (based on history taking and ocular examination): |  |
|  | 1. Red eye | Yes / No |
|  | b. Eyelid edema | Yes / No |
|  | c. Watery eye (epiphora) | Yes / No |
|  | d. Conjunctival secretion | Yes / No |
|  | e. Conjunctival echomosis | Yes / No |
|  | f. Other ocular symptoms, please mention: | _____________________ |
| 5. | Bedside Uncorrected Visual Acuity (UCVA) | ____________ |
| 6. | Ophthalmology diagnosis: (OD/OS/ODS) | 1………………………  2……………………....  3……………………… |
| 7. | Result of supporting examinations:   - Chest CT- Scan - Blood examination   *from medical record | - Data presented / Data not presented - Data presented / Data not presented |
| 8. | Status of Covid-19 | Probable Covid-19 / Confirmed Covid-19 |
| 9. | Result of nasopharyngeal RT-PCR swab test | Positive/ Negative |
| 10. | Date of nasopharyngeal RT-PCR swab taken | ___/___/___ |
| 11. | Result of rapid test COVID-19 blood work | Positive/Negative |
